# Performance of plasma von Willebrand factor in acute traumatic brain injury: relations to severity, CT findings, and outcomes

**DOI:** 10.3389/fnins.2023.1222345

**Published:** 2023-11-23

**Authors:** Rong Zeng, Shaoping Li, Jiangtao Yu, Haoli Ma, Yan Zhao

**Affiliations:** ^1^Emergency Center, Zhongnan Hospital of Wuhan University, Wuhan, Hubei, China; ^2^Department of Biological Repositories, Zhongnan Hospital of Wuhan University, Wuhan, China

**Keywords:** traumatic brain injury, VWF, GCS, GOS, Rotterdam CT score, 6-month mortality

## Abstract

**Background:**

von Willebrand factor (VWF) has been widely recognized as a biomarker for endothelial cell activation in trauma and inflammation. Traumatic brain injury (TBI) is characterized by cerebral vascular injury and subsequent inflammation. The objective of this study was to investigate the correlation between VWF levels and clinical severity, as well as imaging abnormalities, in TBI patients. Additionally, the predictive value of VWF for patient outcomes was assessed.

**Methods:**

We conducted a prospective study to recruit acute TBI patients who were admitted to the emergency department within 24 h. Healthy individuals from the medical examination center were recruited as the control group. This study aimed to compare the accuracy of VWF in discriminating TBI severity and imaging abnormalities with the Glasgow Coma Scale (GCS) and Rotterdam computed tomography (CT) scores. We also analyzed the predictive value of these outcomes using the Glasgow Outcome Scale (GOS) and 6-month mortality.

**Results:**

The plasma concentration of VWF in TBI patients (84.7 ± 29.7 ng/ml) was significantly higher than in healthy individuals (40 ± 8.8 ng/ml). There was a negative correlation between VWF levels and GCS scores, as well as a positive correlation between VWF levels and Rotterdam CT scores. The area under the curve (AUC) for VWF in discriminating mild TBI was 0.76 (95% CI: 0.64, 0.88), and for predicting negative CT findings, it was 0.82 (95% CI: 0.72, 0.92). Meanwhile, the AUC of VWF in predicting mortality within 6 months was 0.70 (95% CI: 0.56, 0.84), and for a GOS score lower 4, it was 0.78 (95% CI: 0.67, 0.88). Combining VWF with either the GCS or Rotterdam CT score improved the prediction ability compared to using VWF alone.

**Conclusion:**

VWF levels were significantly elevated in patients with TBI compared with healthy individuals. Furthermore, VWF levels demonstrated a negative correlation with GCS scores and a positive correlation with Rotterdam CT scores. In terms of predicting mortality, VWF alone was not sufficient, but its predictive power was enhanced when combined with either the Rotterdam CT score or GCS. These findings suggest that VWF may serve as a potential biomarker for assessing the severity and prognosis of TBI patients.

## Introduction

Traumatic brain injury (TBI) is one of the most common diseases in the emergency department and a leading cause of death and disability, imposing a significant economic burden on individuals and society (Hyder et al., 2007). Globally, over 50 million people experience TBI annually, with a high likelihood of TBI incidents occurring throughout their lifetimes (Tang et al., 2020).

TBI progresses rapidly, presents numerous complications, and has a high mortality rate. Therefore, timely and accurate evaluation of TBI patients is very important for distinguishing between those who need emergency surgery and those who need conservative observation (Hon et al., 2019; Vavilala et al., 2019; Al-Hajj et al., 2021; Miller et al., 2021). Accurate diagnosis, triage, and treatment are essential for TBI patients, who are typically stratified based on the Glasgow Coma Scale (GCS), which assesses neurological dysfunction by evaluating eye, verbal, and motor responses. The GCS categorizes TBI severity as severe (GCS 3–8), moderate (GCS 9–12), and mild (GCS 13–15) TBI. However, the GCS has several limitations, often resulting in an inaccurate classification of TBI severity (Stocchetti et al., 2004). Cranial computer tomography (CT) scans are commonly used in the early stages to determine the severity of brain injuries. Although repeated CT scans improve diagnostic accuracy, they also increase the risk of radiation exposure (Brenner and Hall, 2007). Moreover, CT examination may not detect relatively minor lesions (Papa et al., 2012). Although magnetic resonance imaging (MRI) offers higher sensitivity and specificity, it is not suitable for patients with severe conditions requiring continuous mechanical ventilation (Tokshilykova et al., 2020). Its high cost and limited accessibility restrict its use for repeated monitoring of TBI progression. The Glasgow Outcome Scale (GOS) is often used to evaluate clinical outcome variables, including survival and neurological assessment, in TBI patients, but it also has some limitations (Corral et al., 2007; Lu et al., 2012). Therefore, there is a need to explore new methods for detecting changes in the brain structure or function that may have important implications for prognosis (Kurca et al., 2006).

In recent years, there has been an increasing focus on experimental and clinical studies aimed at identifying blood-based biomarkers for the diagnosis and prognostic evaluation of TBI (Anada et al., 2018). Inflammation is one of the main pathophysiological mechanisms of TBI (Abdul-Muneer et al., 2015). The impact or shear force experienced by cerebral blood vessels during TBI can lead to compression, damage, or a cascade of reactions due to brain tissue contusion and edema (Nawashiro et al., 1995; DeWitt and Prough, 2003). These injuries may result in cerebral vessel rupture, hemorrhage, and thrombosis, which can subsequently cause severe consequences such as stroke. Therefore, when addressing TBI, it is crucial to not only focus on the damage to brain tissue itself but also closely monitor cerebrovascular injuries. von Willebrand factor (VWF) serves as a critical factor in promoting platelet recruitment to the site of vascular injury and regulating hemostasis during vascular injury (Xu et al., 2019). Furthermore, VWF has recently been demonstrated to play a role in promoting inflammatory processes and blood–brain barrier (BBB) damage in mice with cerebral hemorrhage (Zhu et al., 2016).

Therefore, our study aims to determine whether there is any difference in plasma VWF levels between TBI patients and healthy individuals. At the same time, the relationship between VWF expression and the severity, imaging abnormalities, and prognosis of acute TBI was studied.

## Methods

### Study design

We prospectively recruited TBI patients who visited emergency department of Zhongnan Hospital of Wuhan University between August 2020 and December 2020. Additionally, healthy individuals from the medical examination center during the same period were recruited as control participants. The primary focus of this study was to explore the effectiveness of VWF in identifying the severity and prognosis of TBI.

### Ethic information

This study was approved by Medical Ethical Committee of Zhongnan Hospital of Wuhan University (approval number 2020121). All procedures were carried out in accordance with the principles outlined in the Code of Ethics of The World Medical Association for experiments involving humans (Declaration of Helsinki) as well as the guidelines for research on health databases (Declaration of Taipei). All data were acquired after informed consent were obtained from the patients.

### Patient inclusion

Consecutive TBI patients who were admitted to the emergency department were recruited for this study. The inclusion criteria were patients aged over 18 years old with acute TBI and no obvious injuries at other sites. However, patients who were pregnant or had a history of hematologic disorders, malignant tumors, uremia, heart failure, severe systemic diseases, chronic TBI, epilepsy, neurological diseases, psychiatric diseases, or intracranial hemorrhage were excluded. Patients who did not undergo brain CT scanning within 24 h after injury were also excluded. Additionally, healthy individuals aged over 18 years old from the medical examination center were recruited as control participants.

### Definition of TBI and severity

Clinical TBI diagnostic criteria included a history of traumatic injury, consciousness alterations, and neurological injury based on CT scanning. The severity assessment of TBI was based solely on the lowest GCS score obtained either at the scene of the accident or in the emergency department. A GCS value of 3–8 was considered severe, 9–12 as moderate, and 13–15 as mild TBI.

### Rotterdam CT score

The Rotterdam CT score is a widely used radiological scoring system for assessing the severity of TBI on CT scans (Huang et al., 2012). In this study, we employed the Rotterdam CT score to evaluate the extent of TBI in our patient cohort. The status of the basal cisterns was assessed and classified into three categories: normal (score 0), compressed (score 1), or absent (score 2). Midline shift was evaluated and categorized as either 0–5 millimeters (score 0) or above 5 millimeters (score 1). The presence or absence of an epidural hematoma was documented as a score of 0 or 1, respectively. Additionally, the presence of traumatic subarachnoid hemorrhage or intraventricular hemorrhage was recorded as a score of 1 accordingly. Finally, one point was added to the total score. Two independent radiologists, who were blinded to the patient's clinical information, reviewed the CT scans and assigned scores based on predefined criteria. To ensure inter-rater reliability, a subset of CT scans was randomly selected and independently assessed by both radiologists. Inter-rater agreement was determined using the intraclass correlation coefficient (ICC). All CT scans were performed using standardized acquisition protocols and interpreted by experienced radiologists. Any discrepancies in the scoring were resolved through discussion and consensus.

### Data collection outcomes

After obtaining informed consent from patients, we collected information on participants and their blood samples. Relevant data, such as age, sex, GCS score, Rotterdam CT score, and GOS score, were extracted from the medical database. The blood samples of the participants were obtained within 1 h of admission. Samples were centrifuged and stored at −80°C for further detection.

### VWF measurements

The plasma concentration of VWF was analyzed by a commercial ELISA kit (CEA833Hu, Cloud-Clone Corp., China) in accordance with the manufacturer's instructions.

### Statistical analysis

Continuous data were presented as mean ± standard error (SD), and categorical data were presented as numbers (percentage). All data were analyzed using GraphPad Prism (Version 8.0.1; GraphPad Software Inc.). Statistical methods, including *t*-tests and Wilcoxon rank-sum tests, were selected based on the characteristics of the data. Pearson's rank correlation was used for correlation analysis. The discriminatory ability of the parameters was assessed by comparing the area under the receiver operating characteristic curve (AUROC). Statistical significance was defined as a two-tailed *P* < 0.05.

## Results

### Characteristics of patients and healthy individuals

**Figure 1:**
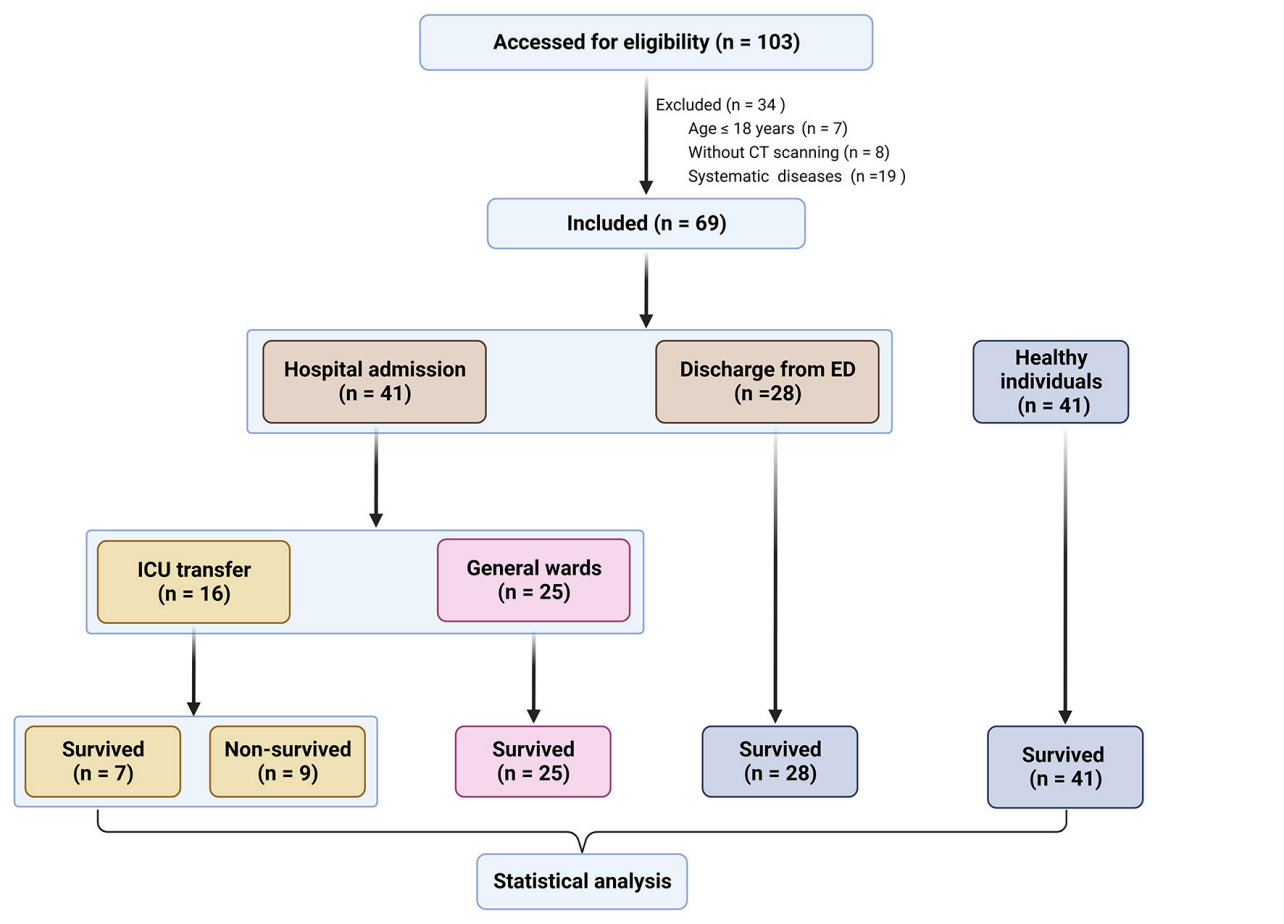
Flowchart of the selection of patients with traumatic brain injury (TBI) and healthy individuals. ED, emergency department; ICU, intensive care unit; CT, computed tomography.

**Figure 2:**
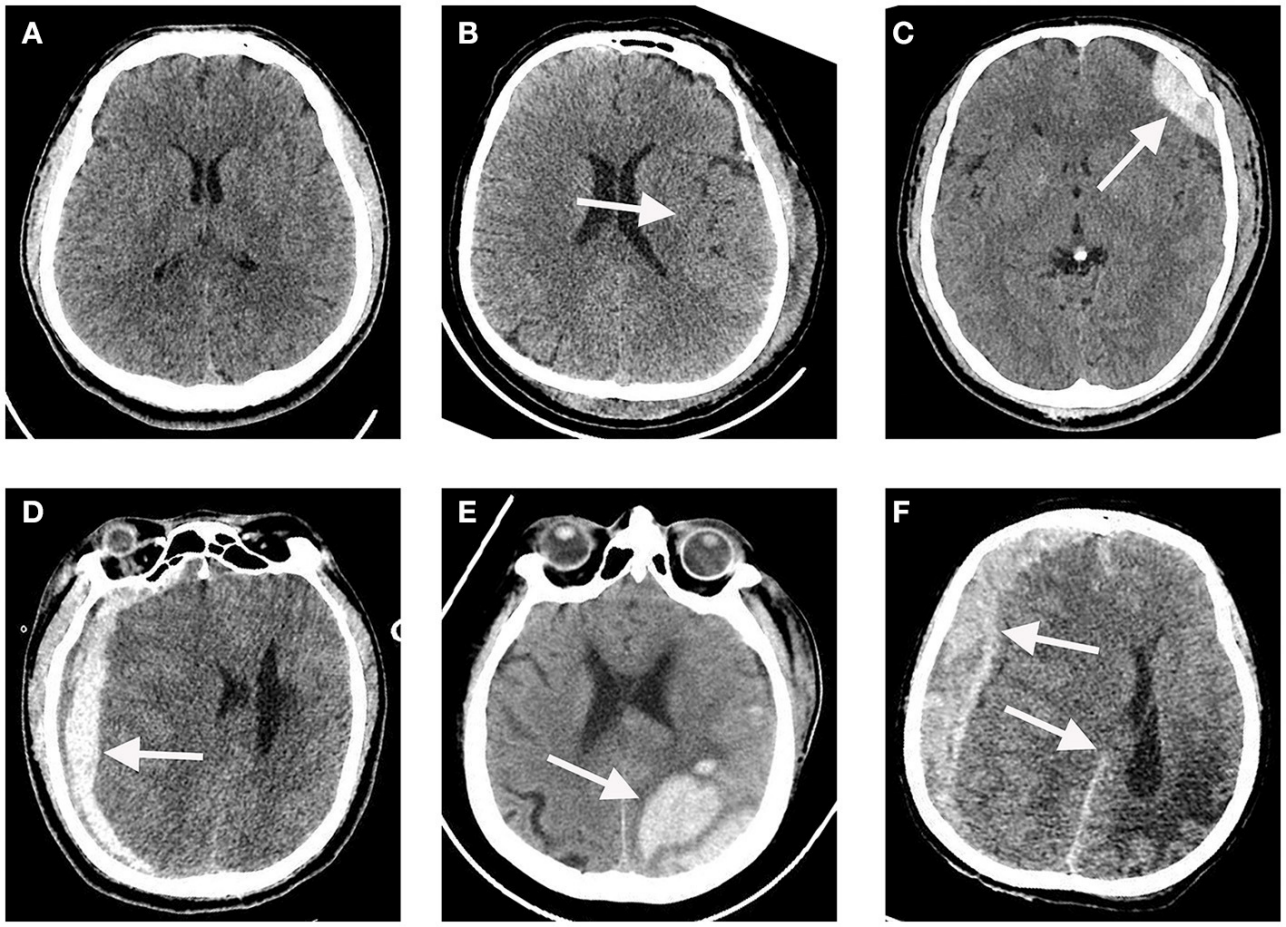
Representative CT images of a traumatic brain injury (TBI) and a normal CT scan. **(A)** Normal CT scan. **(B)** Cerebral contusion. **(C)** Extradural hematoma. **(D)** Subdural hematoma. **(E)** Subarachnoid hemorrhage. **(F)** Hemorrhage with midline shift. The white arrows indicate lesion regions.

A total of 110 participants, consisting of 69 TBI patients and 41 healthy individuals, were included in our study. The flowchart in Figure 1 provides an overview of the patient inclusion process. There was no significant difference in age between the two groups. However, the TBI group had a significantly higher proportion of male patients compared to the control group. The main causes of TBI were vehicle accidents, falls, and assaults. Based on the GCS score, approximately 70%, 6%, and 25% of the patients were categorized as having mild, moderate, and severe TBI, respectively. Furthermore, 37.7% of the patients had a normal CT report (Figure 2A), while the majority (62.3%) exhibited positive CT findings, including contusion (Figure 2B), extradural hematoma (Figure 2C), subdural hematoma (Figure 2D), subarachnoid hemorrhage (Figure 2E), and hemorrhage with midline shift (Figure 2F). In terms of outcomes, 15.9% of the patients had a GOS score of 1–2, indicating a poor outcome, while 84.1% had a GOS score of 3–5, indicating a moderate-to-good outcome. Additionally, the 6-month all-cause mortality rate was 13% (Table 1). In our study, individuals aged 60 and above were classified as elderly. Within the healthy control group and TBI group, the elderly represent 22% and 43.5% of the total population, respectively.

**Table 1:** Demographic and clinical characteristics of the participants.

	**TBI (*n* = 69)**	**Con (*n* = 41)**	***P*-value**
Age, (IQR)	53.0 (37.5–73.5)	50.0 (43–58.5)	0.253
**Sex**, ***n*** **(%)**			
Men	53 (76.8%)	23 (56.1%)	0.023
Women	16 (23.2%)	18 (43.9%)	
Non-elderly (< 60 years)	39 (56.5%)	32 (78%)	
Elderly (≥60 years)	30 (43.5%)	9 (22%)	
**Mechanisms**, ***n*** **(%)**			
Fall	26 (37.7%)		
Assault	11 (15.9%)		
Vehicle accident	32 (46.4%)		
**GCS, (%)**			
Mild 13–15	48 (69.6%)		
Moderate 9–12	4 (5.8%)		
Severe 3–8	17 (24.6%)		
**CT findings**, ***n*** **(%)**			
CT positive scans	43 (62.3%)		
CT negative scans	26 (37.7%)		
**GOS, (%)**			
GOS 1–2	11 (15.9%)		
GOS 3–5	58 (84.1%)		
6-month mortality, *n* (%)	3/23 (13%)		

TBI, traumatic brain injury; GCS, Glasgow Coma Scale; CT, computed tomography; GOS, Glasgow Outcome Scal; VWF, von Willebrand factor.

### Correlation between plasma VWF level and clinical parameters

**Figure 3:**
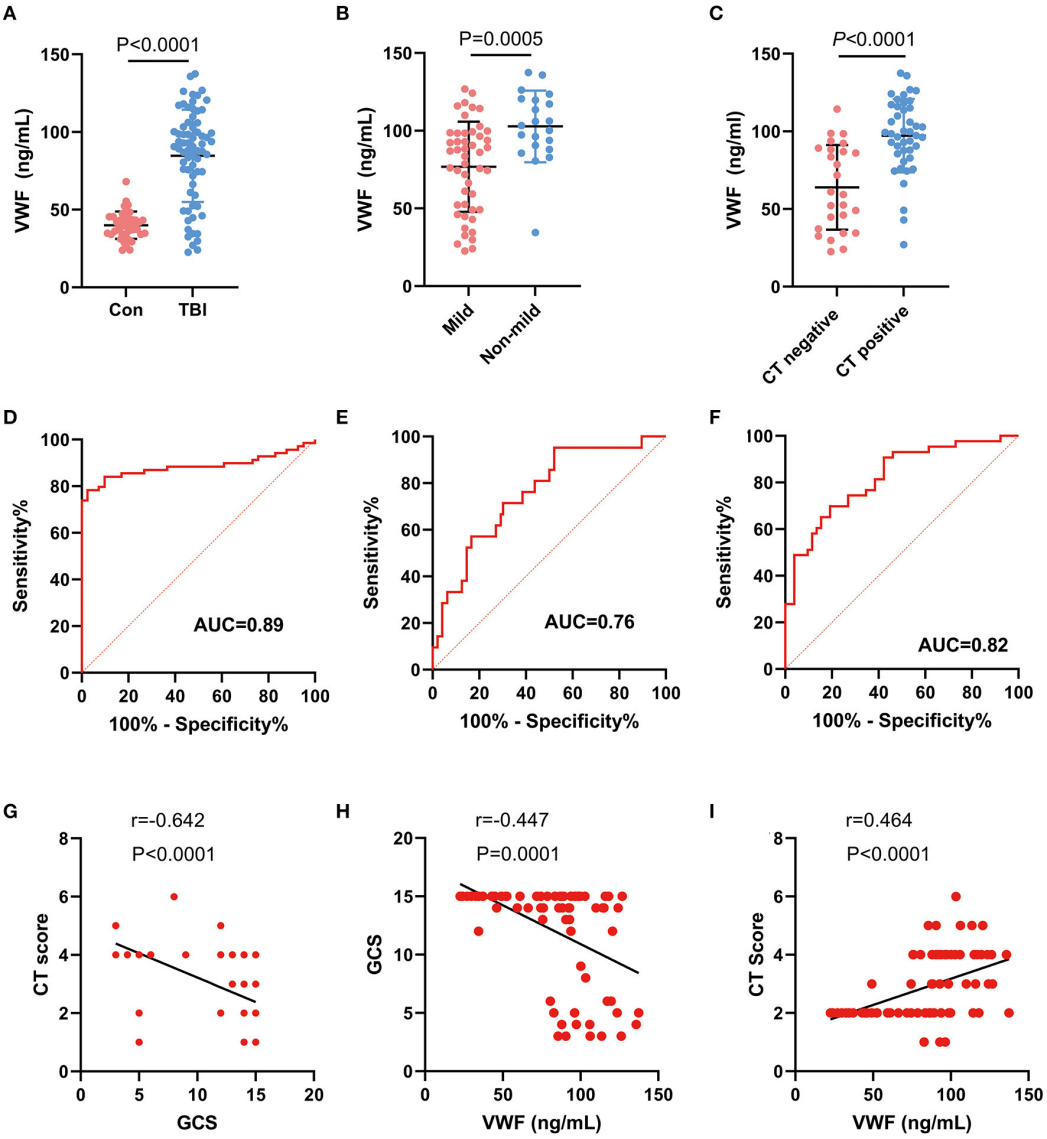
Correlation between plasma VWF level and clinical parameters. **(A)** Difference in plasma VWF concentrations between the control group and TBI patients (*p* < 0.001). **(B)** The level of VWF in patients with mild TBI (76.8 ± 29.0 ng/ml) was significantly lower than that in non-mild TBI (102.8 ± 23.0 ng/ml, *p* < 0.001). **(C)** The level of VWF in patients with negative CT findings (63.9 ± 27.3 ng/ml) was significantly lower than that in patients with positive CT findings (97.2 ± 223.6 ng/ml, *p* < 0.001). **(D)** ROC curve of plasma VWF levels for discriminating TBI patients from healthy individuals. **(E)** ROC curve of plasma VWF levels for discriminating mild TBI from non-mild TBI. **(F)** ROC curve of plasma VWF levels for discriminating negative CT findings from positive CT findings. **(G)** A negative correlation between GCS and CT score (*r* = −0.642, *p* < 0.0001). **(H)** A negative correlation between GCS score and VWF concentration (*r* = −0.45, *p* < 0.0001). **(I)** A positive correlation between VWF and CT score (*r* = 0.46, *p* < 0.0001).

The TBI group (*n* = 69) had a significantly higher plasma concentration of VWF (84.68 ± 29.72 ng/ml) compared with the healthy individuals (*n* = 41) (39.97 ± 8.79 ng/ml, *p* < 0.0001, Figure 3A). The *post-hoc* power after data collection was 1. Furthermore, the level of VWF in patients with mild TBI (76.8 ± 29.0 ng/ml) or negative CT findings (63.9 ± 27.3 ng/ml) was significantly lower than that in non-mild TBI (102.8 ± 23.0 ng/ml) or positive CT findings (97.2 ± 223.6 ng/ml, all *p* < 0.001, Figures 3B, C). Among TBI patients, a VWF level of 78.26 ng/mL had the highest discriminatory power to differentiate TBI patients from healthy individuals (AUC, 0.89, sensitivity, 67%, specificity, 98%, Figure 3D). Moreover, VWF had an AUC of 0.76 (95% CI: 0.64, 0.88, Figure 3E) for discriminating mild TBI and 0.82 (95% CI: 0.72, 0.92, Figure 3F) for predicting negative CT findings. Furthermore, we analyzed the correlation between VWF, GCS, and CT scores in TBI patients. We found a negative correlation between GCS and CT score (r = −0.642, *p* < 0.0001, Figure 3G), indicating that patients with lower GCS scores were more likely to have severe brain structural damage detected by CT scanning. Additionally, VWF was negatively correlated with the GCS score (r = −0.447, *p* < 0.0001, Figure 3H) and significantly positively correlated with the CT score (*r* = 0.464, *p* < 0.0001, Figure 3I). Additionally, within the TBI group and the healthy control group, we separately examined the potential impact of age and gender on VWF levels. Our results showed that neither age nor gender had any significant influence on VWF levels (Supplementary Figure 1).

### VWF improves prediction of clinical recovery

There were eight patients who died in the hospital and one patient who died within 6 months of discharge. The predictive ability of VWF for 6-month mortality was evaluated, and it was found to have an area under the curve (AUC) of 0.70. However, this did not reach the threshold for statistical significance (Table 2, *p* = 0.06). When combined with either CT score or GCS, VWF showed improved predictive ability (*p* < 0.0001). Given that a GOS score of ≤ 4 is closely associated with morbidity, the discriminatory power of VWF in predicting patients with a GOS of ≤ 4 was also analyzed. VWF demonstrated an AUC of 0.77 (*p* < 0.0001) for GOS ≤ 4. Similarly, the combined use of VWF with either a GCS or CT score showed improved predictive ability. These findings indicate that VWF alone is not sufficient for predicting 6-month mortality, but it can effectively identify patients at high risk of morbidity following TBI. Therefore, combining VWF with either the CT score or the GCS significantly enhances the predictive ability.

**Table 2:** Discrimination power of the VWF, CT score, and GCS for mortality and GOS≤4 in TBI patients.

	**Mortality**			**GOS** ≤ **4**	
	**AUC**	***P*** **-value**		**AUC**	***P*** **-value**
GCS	0.13	*P* < 0.0001	GCS	0.14	*P* < 0.0001
CT Score	0.86	*P* < 0.0001	CT Score	0.83	*P* < 0.0001
VWF	0.70	*P* = 0.06	VWF	0.77	*P* < 0.0001
GCS + VWF	0.87	*P* < 0.0001	GCS + VWF	0.90	*P* < 0.0001
CT + VWF	0.88	*P* < 0.0001	CT + VWF	0.87	*P* < 0.0001
CT + GCS	0.91	*P* < 0.0001	CT + GCS	0.90	*P* < 0.0001
VWF + CT + GCS	0.90	*P* < 0.0001	VWF + CT + GCS	0.91	*P* < 0.0001

VWF, von Willebrand factor; CT, computed tomography; GCS, Glasgow Coma Scale; GOS, Glasgow Outcome Scale; TBI, traumatic brain injury; AUC, area under the receiver operating characteristic curve.

## Discussion

A blood-based biomarker associated with pathophysiological mechanisms can aid in the identification of TBI, prediction and monitoring of recovery post-injury, and guidance for clinical treatment (Lippa et al., 2020). TBI inevitably results in vascular injury or blood flow disorders; hence, it is essential to explore biomarkers associated with TBI-related vascular injury. Previous clinical studies have shown that VWF, released from injured endothelial cells and activated platelets during acute injury, significantly elevates plasma VWF levels (Yokota et al., 2002; Tang et al., 2013). However, there is a paucity of studies investigating whether VWF can differentiate between TBI patients and healthy individuals or serve as a biomarker for evaluating TBI severity.

In this study, plasma levels of VWF were measured in patients with acute TBI and healthy individuals. The study aimed to analyze the relationship between VWF and the GCS, Rotterdam CT score, and GOS. The findings revealed that patients with acute TBI had higher plasma VWF levels compared to healthy individuals. Additionally, VWF levels were negatively correlated with GCS scores and significantly positively correlated with Rotterdam CT scores. While VWF alone was not sufficient to predict mortality, its predictive power improved when combined with either the Rotterdam CT score or GCS. Furthermore, our study demonstrates that VWF levels have the capability to differentiate between patients with unfavorable outcomes (GOS 1–4) and those with favorable outcomes (GOS 5) subsequent to TBI. Previous research has demonstrated that VWF serves as a sensitive indicator of endothelial cell activation (Ward et al., 2020). In the context of traumatic injury, an appropriate concentration of VWF can effectively control bleeding and reduce hemorrhage (Holcomb et al., 2015; Ng et al., 2015). Additionally, a high concentration of VWF independently increases the risk of deep vein thrombosis formation (Setiawan et al., 2020). The excessive release of VWF can lead to the formation of emboli within blood vessels, resulting in compromised blood flow and exacerbated ischemic injury (Nguyen et al., 2008). VWF is promising as a monitoring marker of systemic injury in brain trauma or critical diseases (Plautz et al., 2020). Our findings provide evidence of a significant correlation between the concentration of VWF and both the severity and prognosis of acute TBI. These findings are consistent with the results reported in previous clinical studies (De Oliveira et al., 2007; Sandsmark et al., 2019). In TBI, rupture of blood vessels and impaired endothelial cells can lead to increased release of VWF, contributing to platelet aggregation and thrombus formation. This can result in microcirculatory disturbances and local ischemia, further exacerbating brain injury (Lu et al., 2004; Wu et al., 2018). Moreover, TBI-induced endothelial injury and an inflammatory response can increase vascular permeability. VWF, through its binding to receptors on the endothelial cell surface, facilitates vascular leakage in TBI (Zhu et al., 2016; Aymé et al., 2017; Wu et al., 2018). Excessive VWF may promote the extravasation of blood components into brain tissue, leading to edema and an inflammatory response. In addition to its roles in coagulation and vascular biology, VWF is also involved in modulating the inflammatory process (Chauhan et al., 2008). It can interact with inflammatory mediators, influencing the activation and migration of inflammatory cells. In TBI, VWF may impact the development and recovery of injury by modulating the inflammatory response. However, further investigation is necessary to establish VWF as a reliable biomarker for the diagnosis and prognosis of acute TBI.

This study has several limitations. First, due to the location of our department in the emergency department, our study focused primarily on assessing outcomes during the ED visit or initial presentation. Long-term follow-up and evaluation of VWF levels, GCS scores, and detailed prognosis were not conducted. Second, the diagnostic and predictive value of VWF in diseases other than TBI or in combination with other injuries remains unknown. Third, the findings of this study are limited to a single location and a relatively small sample size, which may restrict the generalizability of the results. Finally, further research is necessary to identify additional blood biomarkers and enhance the accuracy and reliability of TBI diagnosis and prognosis.

## Conclusion

Plasma VWF concentrations were found to be significantly higher in patients with acute TBI compared to healthy individuals. In patients with acute TBI, VWF levels showed a negative correlation with GCS scores and a positive correlation with CT scores and TBI severity. However, the ability of VWF alone to predict mortality in acute TBI patients was limited, and its predictive value was enhanced when combined with Rotterdam CT scores or GCS scores. Therefore, VWF may serve as a potential biomarker for the diagnosis of acute TBI.

## Data availability statement

The datasets presented in this study can be found in online repositories. The names of the repository/repositories and accession number(s) can be found in the article/Supplementary material.

## Ethics statement

The studies involving humans were approved by Medical Ethics Committee of Zhongnan Hospital of Wuhan University. The studies were conducted in accordance with the local legislation and institutional requirements. The participants provided their written informed consent to participate in this study. Written informed consent was obtained from the individual(s) for the publication of any potentially identifiable images or data included in this article.

## Author contributions

RZ, YZ, and HM conceived and designed the experiments. SL performed the data analysis. RZ and SL wrote the manuscript. All authors reviewed and approved the manuscript.

## References

[B1] Abdul-Muneer P. M. Chandra N. Haorah J. (2015). Interactions of oxidative stress and neurovascular inflammation in the pathogenesis of traumatic brain injury. Mol. Neurobiol. 51, 966–979. 10.1007/s12035-014-8752-3 24865512 PMC9420084

[B2] Al-Hajj S. Hammoud Z. Colnaric J. Ataya M. Macaron M. M. Kadi K.. (2021). Characterization of traumatic brain injury research in the middle east and north africa region: a systematic review. Neuroepidemiology. 10, 1–12. 10.1159/000511554 33567436

[B3] Anada R. P. Wong K. T. Jayapalan J. J. Hashim O. H. Ganesan D. (2018). Panel of serum protein biomarkers to grade the severity of traumatic brain injury. Electrophoresis 39, 2308–2315. 10.1002/elps.201700407 29570807

[B4] Aymé G. Adam F. Legendre P. Bazaa A. Proulle V. Denis C. V.. (2017). A novel single-domain antibody against von willebrand factor A1 domain resolves leukocyte recruitment and vascular leakage during inflammation-brief report. Arterioscler. Thromb Vasc. Biol. 37, 1736–1740. 10.1161/ATVBAHA.117.309319 28642239

[B5] Brenner D. J. Hall E. J. (2007). Computed tomography–an increasing source of radiation exposure. N. Engl. J. Med. 357, 2277–2284. 10.1056/NEJMra072149 18046031

[B6] Chauhan A. K. Kisucka J. Brill A. Walsh M. T. Scheiflinger F. Wagner D. D. (2008). ADAMTS13: a new link between thrombosis and inflammation. J. Exp. Med. 205, 2065–2074. 10.1084/jem.20080130 18695007 PMC2526201

[B7] Corral L. Ventura J. L. Herrero J. I. Monfort J. L. Juncadella M. Gabarrós A.. (2007). Improvement in GOS and GOSE scores 6 and 12 months after severe traumatic brain injury. Brain Inj. 21, 1225–1231. 10.1080/02699050701727460 18236198

[B8] De Oliveira C. O. Reimer A. G. Da Rocha A. B. Grivicich I. Schneider R. F. Roisenberg I.. (2007). Plasma von Willebrand factor levels correlate with clinical outcome of severe traumatic brain injury. J. Neurotrauma 24, 1331–1338. 10.1089/neu.2006.0159 17711394

[B9] DeWitt D. S. Prough D. S. (2003). Traumatic cerebral vascular injury: the effects of concussive brain injury on the cerebral vasculature. J. Neurotrauma 20, 795–825. 10.1089/089771503322385755 14577860

[B10] Holcomb J. B. Tilley B. C. Baraniuk S. Fox E. E. Wade C. E. Podbielski J. M.. (2015). Transfusion of plasma, platelets, and red blood cells in a 1:1:1 vs a 1:1:2 ratio and mortality in patients with severe trauma: the PROPPR randomized clinical trial. JAMA 313, 471–482. 10.1001/jama.2015.12 25647203 PMC4374744

[B11] Hon K. L. Leung A. K. C. Torres A. R. (2019). Concussion: a global perspective. Semin Pediatr. Neurol. 30, 117–127. 10.1016/j.spen.2019.03.017 31235013

[B12] Huang Y. H. Deng Y. H. Lee T. C. Chen W. F. (2012). Rotterdam computed tomography score as a prognosticator in head-injured patients undergoing decompressive craniectomy. Neurosurgery 71, 80–85. 10.1227/NEU.0b013e3182517aa1 22382208

[B13] Hyder A. A. Wunderlich C. A. Puvanachandra P. Gururaj G. Kobusingye O. C. (2007). The impact of traumatic brain injuries: a global perspective. NeuroRehabilitation 22, 341–353. 10.3233/NRE-2007-22502 18162698

[B14] Kurca E. Sivák S. Kucera P. (2006). Impaired cognitive functions in mild traumatic brain injury patients with normal and pathologic magnetic resonance imaging. Neuroradiology 48, 661–669. 10.1007/s00234-006-0109-9 16786351

[B15] Lippa S. M. Werner J. K. Miller M. C. Gill J. M. Diaz-Arrastia R. Kenney K. (2020). Recent advances in blood-based biomarkers of remote combat-related traumatic brain injury. Curr. Neurol. Neurosci. Rep. 20, 54. 10.1007/s11910-020-01076-w 32984931

[B16] Lu D. Mahmood A. Goussev A. Qu C. Zhang Z. G. Chopp M. (2004). Delayed thrombosis after traumatic brain injury in rats. J. Neurotrauma 21, 1756–1766. 10.1089/neu.2004.21.1756 15684767

[B17] Lu J. Marmarou A. Lapane K. L. (2012). Impact of GOS misclassification on ordinal outcome analysis of traumatic brain injury clinical trials. J. Neurotrauma 29, 719–726. 10.1089/neu.2010.1746 21815785 PMC3303101

[B18] Miller G. F. Daugherty J. Waltzman D. Sarmiento K. (2021). Predictors of traumatic brain injury morbidity and mortality: examination of data from the national trauma data bank: predictors of TBI morbidity and mortality. Injury 52, 1138–1144. 10.1016/j.injury.2021.01.042 33551263 PMC8107124

[B19] Nawashiro H. Shima K. Chigasaki H. (1995). Immediate cerebrovascular responses to closed head injury in the rat. J. Neurotrauma 12, 189–197. 10.1089/neu.1995.12.189 7629865

[B20] Ng C. Motto D. G. Di Paola J. (2015). Diagnostic approach to von Willebrand disease. Blood 125, 2029–2037. 10.1182/blood-2014-08-528398 25712990 PMC4375103

[B21] Nguyen T. C. Han Y. Y. Kiss J. E. Hall M. W. Hassett A. C. Jaffe R.. (2008). Intensive plasma exchange increases a disintegrin and metalloprotease with thrombospondin motifs-13 activity and reverses organ dysfunction in children with thrombocytopenia-associated multiple organ failure. Crit. Care Med. 36, 2878–2887. 10.1097/CCM.0b013e318186aa49 18828196 PMC2772176

[B22] Papa L. Lewis L. M. Falk J. L. Zhang Z. Silvestri S. Giordano P.. (2012). Elevated levels of serum glial fibrillary acidic protein breakdown products in mild and moderate traumatic brain injury are associated with intracranial lesions and neurosurgical intervention. Ann. Emerg. Med. 59, 471–483. 10.1016/j.annemergmed.2011.08.021 22071014 PMC3830977

[B23] Plautz W. E. Matthay Z. A. Rollins-Raval M. A. Raval J. S. Kornblith L. Z. Neal M. D. (2020). Von Willebrand factor as a thrombotic and inflammatory mediator in critical illness. Transfusion. 60, S158–S166. 10.1111/trf.15667 32478907 PMC9053104

[B24] Sandsmark D. K. Bogoslovsky T. Qu B. X. Haber M. Cota M. R. Davis C.. (2019). Changes in plasma von willebrand factor and cellular fibronectin in MRI-defined traumatic microvascular injury. Front. Neurol. 10, 246. 10.3389/fneur.2019.00246 30972003 PMC6445052

[B25] Setiawan B. Permatadewi C. O. de Samakto B. Bugis A. Naibaho R. M. Pangarsa E. A.. (2020). Von Willebrand factor:antigen and ADAMTS-13 level, but not soluble P-selectin, are risk factors for the first asymptomatic deep vein thrombosis in cancer patients undergoing chemotherapy. Thromb J. 18, 33. 10.1186/s12959-020-00247-6 33292287 PMC7659107

[B26] Stocchetti N. Pagan F. Calappi E. Canavesi K. Beretta L. Citerio G.. (2004). Inaccurate early assessment of neurological severity in head injury. J. Neurotrauma 21, 1131–1140. 10.1089/neu.2004.21.1131 15453984

[B27] Tang N. Yin S. Sun Z. Pan Y. (2013). Time course of soluble P-selectin and von Willebrand factor levels in trauma patients: a prospective observational study. Scand. J. Trauma Resusc. Emerg. Med. 21, 70. 10.1186/1757-7241-21-70 24034700 PMC3847632

[B28] Tang Y. L. Fang L. J. Zhong L. Y. Jiang J. Dong X. Y. Feng Z. (2020). Hub genes and key pathways of traumatic brain injury: bioinformatics analysis and in vivo validation. Neural Regen. Res. 15, 2262–2269. 10.4103/1673-5374.284996 32594047 PMC7749465

[B29] Tokshilykova A. B. Sarkulova Z. N. Kabdrakhmanova G. B. Utepkaliyeva A. P. Tleuova A. S. Satenov Z. K. (2020). Neuron-specific markers and their correlation with neurological scales in patients with acute neuropathologies. J. Mol. Neurosci. 70, 1267–1273. 10.1007/s12031-020-01536-5 32350763

[B30] Vavilala M. King M. Yang J. (2019). The Pediatric Guideline Adherence and Outcomes (PEGASUS) programme in severe traumatic brain injury: a single-centre hybrid implementation and effectiveness study. Lancet Child Adolesc. Health 3, 23–34. 10.1016/S2352-4642(18)30341-9 30473440 PMC6301024

[B31] Ward S. E. Curley G. F. Lavin M. Fogarty H. Karampini E. McEvoy N. L.. (2020). Von Willebrand factor propeptide in severe coronavirus disease 2019 (COVID-19): evidence of acute and sustained endothelial cell activation. Br. J. Haematol. 192, 714–719. 10.1111/bjh.17273 33326604

[B32] Wu Y. Liu W. Zhou Y. Hilton T. Zhao Z. Liu W.. (2018). von Willebrand factor enhances microvesicle-induced vascular leakage and coagulopathy in mice with traumatic brain injury. Blood 132, 1075–1084. 10.1182/blood-2018-03-841932 29941674 PMC6128082

[B33] Xu E. R. von Bülow S. Chen P. C. Lenting P. J. Kolšek K. Aponte-Santamaría C.. (2019). Structure and dynamics of the platelet integrin-binding C4 domain of von Willebrand factor. Blood 133, 366–376. 10.1182/blood-2018-04-843615 30305279 PMC6450055

[B34] Yokota H. Naoe Y. Nakabayashi M. Unemoto K. Kushimoto S. Kurokawa A.. (2002). Cerebral endothelial injury in severe head injury: the significance of measurements of serum thrombomodulin and the von Willebrand factor. J. Neurotrauma 19, 1007–1015. 10.1089/089771502760341929 12482114

[B35] Zhu X. Cao Y. Wei L. Cai P. Xu H. Luo H.. (2016). von Willebrand factor contributes to poor outcome in a mouse model of intracerebral haemorrhage. Sci. Rep. 6, 35901. 10.1038/srep35901 27782211 PMC5080593

