# Supplementary figures and images for: Performance of plasma von Willebrand factor in acute traumatic brain injury: relations to severity, CT findings, and outcomes
Source: Front Neurosci. 2023 Nov 23;17:1222345. doi: 10.3389/fnins.2023.1222345 (PMC10706470; doi:10.3389/fnins.2023.1222345)

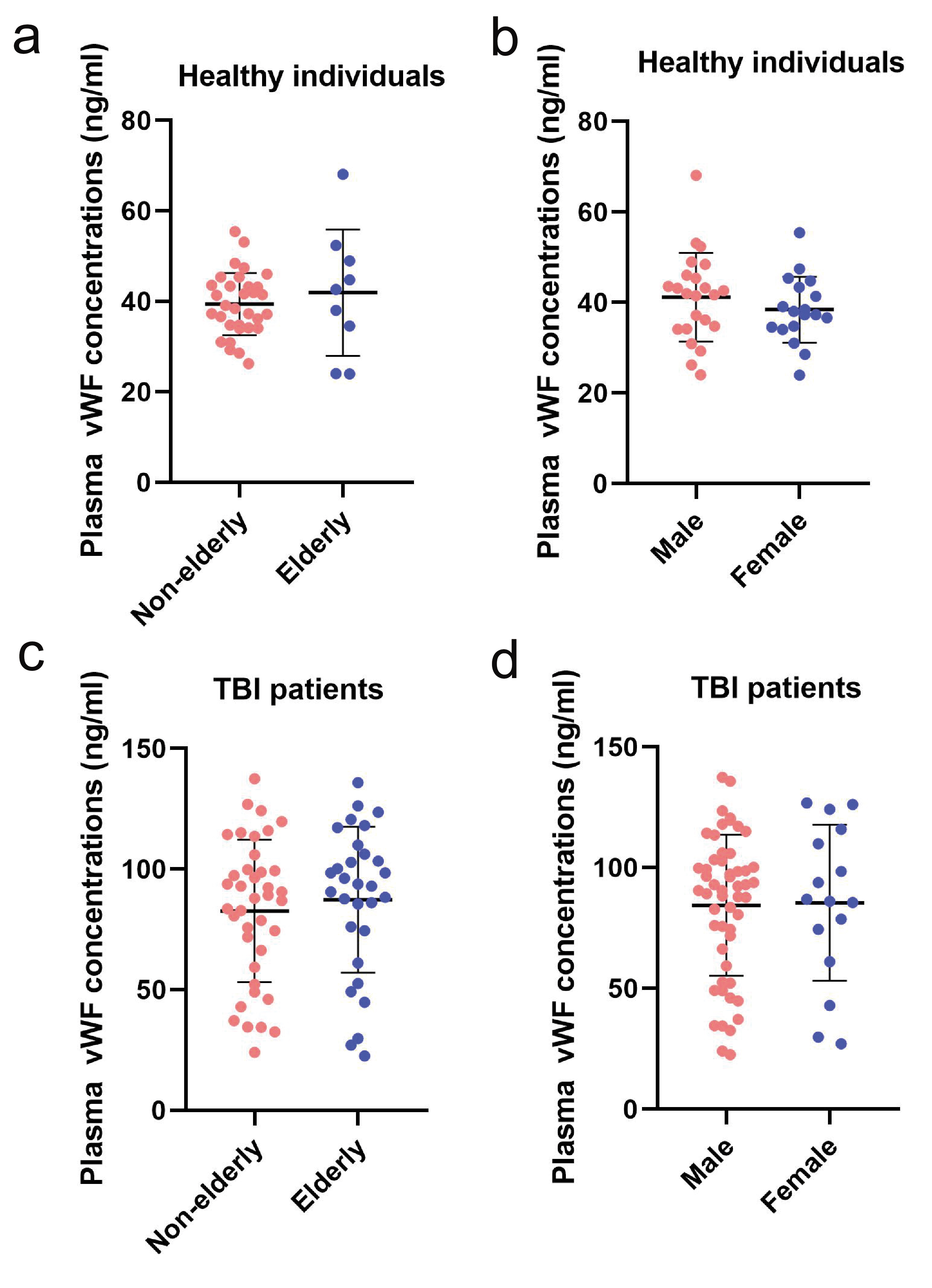

Supplement: Supplementary file 1 [file Image_1.JPEG]
